# CRISPR/Cas9-targeted mutagenesis of the tomato susceptibility gene *PMR4* for resistance against powdery mildew

**DOI:** 10.1186/s12870-020-02497-y

**Published:** 2020-06-19

**Authors:** Miguel I. Santillán Martínez, Valentina Bracuto, Eleni Koseoglou, Michela Appiano, Evert Jacobsen, Richard G. F. Visser, Anne-Marie A. Wolters, Yuling Bai

**Affiliations:** Plant Breeding, Wageningen University & Research, Droevendaalsesteeg 1, 6708 PB Wageningen, The Netherlands

**Keywords:** CRISPR/Cas9, Targeted mutagenesis, *PMR4*, Powdery mildew, Susceptibility gene

## Abstract

**Background:**

The development of CRISPR/Cas9 technology has facilitated targeted mutagenesis in an efficient and precise way. Previously, RNAi silencing of the susceptibility (*S*) gene *PowderyMildewResistance 4* (*PMR4*) in tomato has been shown to enhance resistance against the powdery mildew pathogen *Oidium neolycopersici* (*On*).

**Results:**

To study whether full knock-out of the tomato *PMR4* gene would result in a higher level of resistance than in the RNAi-silenced transgenic plants we generated tomato *PMR4* CRISPR mutants. We used a CRISPR/Cas9 construct containing four single-guide RNAs (sgRNAs) targeting the tomato *PMR4* gene to increase the possibility of large deletions in the mutants. After PCR-based selection and sequencing of transformants, we identified five different mutation events, including deletions from 4 to 900-bp, a 1-bp insertion and a 892-bp inversion. These mutants all showed reduced susceptibility to *On* based on visual scoring of disease symptoms and quantification of relative fungal biomass. Histological observations revealed a significantly higher occurrence of hypersensitive response-like cell death at sites of fungal infection in the *pmr4* mutants compared to wild-type plants. Both haustorial formation and hyphal growth were diminished but not completely inhibited in the mutants.

**Conclusion:**

CRISPR/Cas-9 targeted mutagenesis of the tomato *PMR4* gene resulted in mutants with reduced but not complete loss of susceptibility to the PM pathogen *On.* Our study demonstrates the efficiency and versatility of the CRISPR/Cas9 system as a powerful tool to study and characterize *S*-genes by generating different types of mutations.

## Background

Powdery mildew (PM) in tomato, caused by the obligate biotrophic fungus *Oidium neolycopersici* (*On*), is a world-wide disease that threatens the production of greenhouse- and field-grown tomatoes [1, 2]. Over the last few decades, research focused on breeding for resistance against PM in tomato has resulted in the identification of five dominant resistance (*R*) genes (*Ol-*genes) from wild tomato species [3]. These genes were introgressed into the susceptible tomato cultivar Moneymaker and near-isogenic lines (NILs) were made [4]. Histological studies of these NILs after powdery mildew infection has allowed the identification of two different types of host responses associated with resistance: unicellular hypersensitive response (HR) in *Ol-4* and *Ol-6*, leading to complete resistance against PM; and slow multicellular HR in *Ol-1*, *Ol-3* and *Ol-5*, leading to incomplete resistance [4–6].

In addition to these dominantly-inherited resistance genes a recessive gene named *ol-2* was identified [7]. This is a loss-of-function mutant allele of the powdery mildew susceptibility (*S*) gene, *MILDEW RESISTANCE LOCUS O* (*MLO)* [7]. In homozygous state this *ol-2* allele confers broad-spectrum resistance to different PM species by inducing papilla formation and callose deposition, which block fungal development at penetration stage [6]. The loss-of-function allele of *MLO* is one of the best studied examples of recessively-inherited resistance against PMs [8]. In recent years, many other examples of resistance conferred by the impairment of *S* genes have been described in different pathosystems [9]. The use of such *S* genes in plant breeding, due to their broad-spectrum and potentially durable resistance characteristics, represent a promising alternative to the introgression of *R* genes that has been driving traditional resistance breeding [9–11].

In Arabidopsis, several mutants showing resistance against the adapted PM pathogen *Golovinomyces cichoracearum* have been identified after screening an EMS population [12]. These mutants were named *powdery mildew resistant 1* (*pmr1*) to *pmr4*. The *pmr4* mutant showed resistance not only against *G. cichoracearum*, but also against *G. orontii* and downy mildew *Hyaloperonospora arabidopsidis*. *PMR4* was shown to be the callose synthase gene At4g03550, also known as *GLUCAN SYNTHASE-LIKE 5* (*GSL5)* or *CALLOSE SYNTHASE 12* (*CalS12*) [13, 14]. The *GSL*/*CalS* gene family comprises 12 genes in Arabidopsis [15–17]. The callose synthase encoded by *PMR4* is responsible for the production of callose in response to biotic and abiotic stresses [14]. Both the *pmr4* and a *gsl5* mutant (homozygous for a T-DNA insertion in the *PMR4* gene) showed almost complete lack of callose in infected leaves [12, 13]. Histological analyses proved that these mutants form papillae after PM infection, even in the absence of callose [13, 14].

It would seem strange that mutant plants lacking callose in the papillae formed at attempted penetration sites show PM resistance, especially because Arabidopsis plants overexpressing *PMR4* show complete penetration resistance against *G. cichoracearum* [18, 19]. This latter resistance is based on increased callose deposition at infection sites, which acts as a physical barrier against PM-secreted cell wall hydrolases [19].

However, the *pmr4* mutant develops lesions reminiscent of hypersensitive cell death after PM infection [14]. This cell death likely results from activation of the salicylic acid (SA) signal transduction pathway as genes in this pathway were upregulated in the *pmr4* mutant compared to the wild type control, and blocking the SA pathway in the *pmr4* mutant (using double mutants) was enough to restore full susceptibility to PM [14]. Probably, PMR4 is not only involved in callose synthesis at specific sites after attempted fungal penetration, but also negatively controls the SA-associated defense pathway [20]. In plants overexpressing *PMR4* the SA pathway is not induced [18]. Thus, different mechanisms are involved in resistance by overexpressing or knocking-out the *PMR4* gene.

To investigate whether the PMR4 function is conserved in other plants species than Arabidopsis, the closest tomato ortholog of *PMR4* (Solyc07g053980; *SlPMR4*) was silenced by RNAi [21]. Transgenic plants in which the gene was well-silenced showed enhanced resistance against the tomato PM pathogen (*On*) and a slight reduction in plant size when compared to the non-silenced controls [21]. These RNAi plants still showed a low level of expression of the *SlPMR4* gene. Knock-down-based methods for characterization of *S* genes, such as RNAi or virus-induced gene silencing (VIGS), usually result in residual expression of the targeted genes, which typically causes partial phenotypes complicating functional analysis of the genes [22]. Mutations resulting in full knock-out of gene function sometimes produce a stronger phenotype than knock-down transgenic plants obtained by RNAi silencing [23, 24] . We set out to produce tomato *SlPMR4* mutants to investigate whether complete resistance against *On* could be obtained in this way, compared with the substantial but not complete resistance in the RNAi transgenic plants [21] . For this, targeted mutagenesis is the preferred method. The generation of precise, stable and heritable knock-out alleles of *S* genes is now possible with the development of the clustered regularly interspaced short palindromic repeats (CRISPR) technology. This technology has already been used to generate PM-resistant *slmlo1* tomato mutants [25], and PM-resistant wheat by the simultaneous modification of the three *EDR1* homologues [26]. In addition, host resistance in other pathosystems has been achieved using CRISPR-induced targeted mutation [27, 28]. In this study, we report the successful generation and characterization of five different mutation events via CRISPR/Cas9 in the tomato ortholog of the *PMR4* gene. Further, we show that PM resistance in these *pmr4* mutants is associated with cell death upon PM infection.

## Results

### CRISPR/Cas-9-targeted mutagenesis of *SlPMR4*

**Fig. 1:**
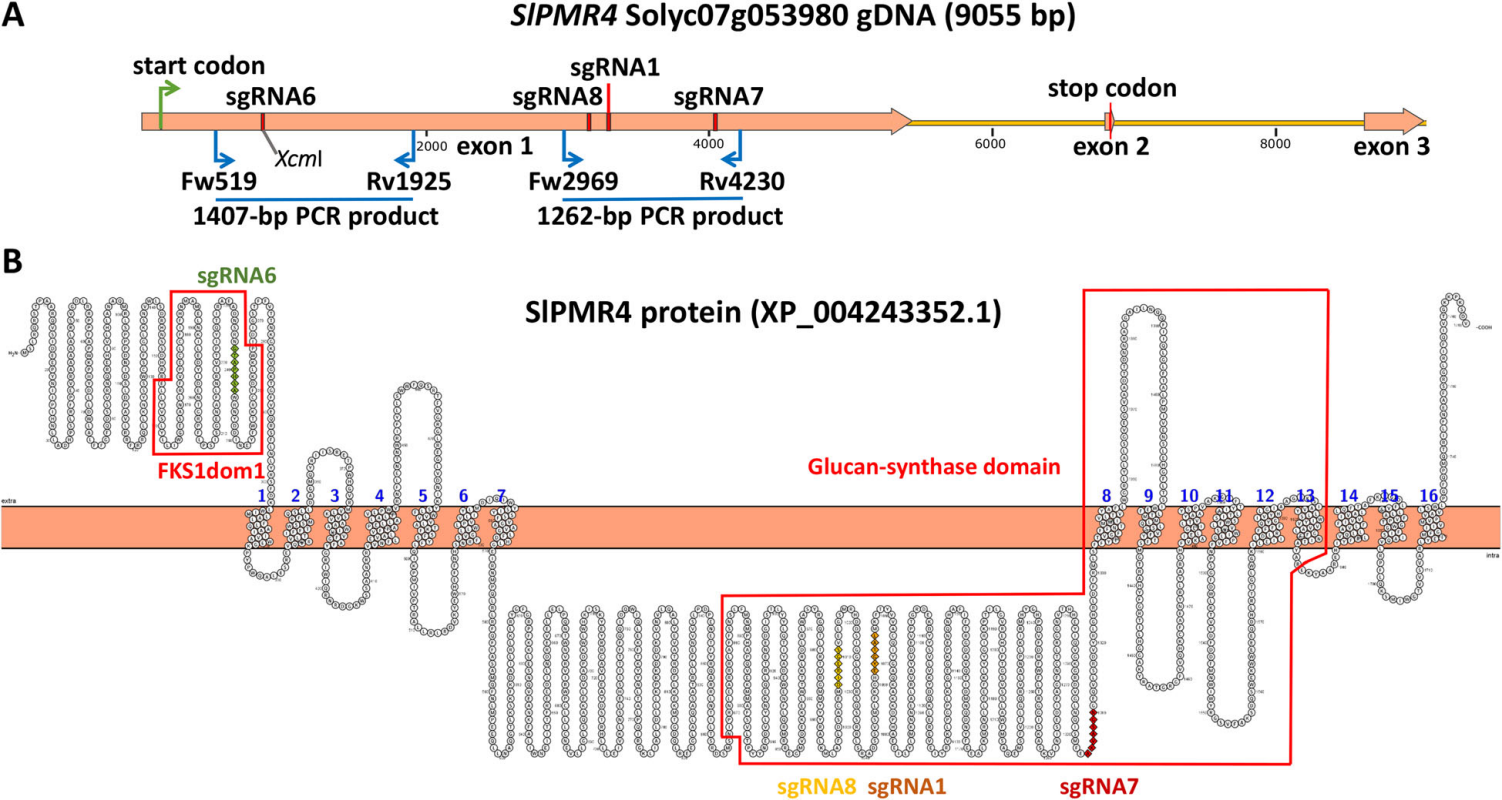
Position of target sites of the sgRNAs in *SlPMR4*. **a.** Representation of genomic sequence of *SlPMR4* showing the position of four single guide RNAs (sgRNAs) that were designed to produce knock-out mutants of *SlPMR4*. PCR primers Fw519 + Rv1925 and Fw2969 + Rv4230 were used to identify mutants. **b.** PROTTER [31] representation of the SlPMR4 protein. sgRNA6 (green) is located in the N-terminal FKS1dom1 domain of the protein; sgRNA8 (yellow), sgRNA1 (orange) and sgRNA7 (red) target sites in the intracellular part of the Glucan-synthase domain

To produce mutants of the tomato *PMR4* ortholog (Solyc07g053980) [21] a single CRISPR/Cas-9 construct containing four sgRNAs (sgRNA6, sgRNA8, sgRNA1 and sgRNA7; Supplementary Table 1) was made. We used four sgRNAs in order to increase the chances of obtaining mutants with large deletions [29, 30] . The position of the four sgRNAs in the *SlPMR4* genomic sequence and in the predicted SlPMR4 protein can be seen in Fig. 1. *PMR4* contains two known protein domains according to NCBI Conserved Domains (https://www.ncbi.nlm.nih.gov/cdd/): FKS1dom1 [32] and the Glucan-synthase domain [33]. One of the four sgRNAs (sgRNA6) targets the FKS1dom1 domain in the N-terminal region. The three remaining sgRNAs target the intracellular part of the Glucan-synthase domain. The *SlPMR4* CRISPR/Cas9 construct was used to transform the susceptible tomato cultivar Moneymaker (MM).

In total, 37 primary transformants (T1) were obtained and analyzed. Individual T1 plants containing mutations within the *SlPMR4* gene were selected via PCR amplification. Two different primer combinations were used: primers Fw519 + Rv1925 flanking sgRNA6 and primers Fw2969 + Rv4230 flanking sgRNA8, sgRNA1 and sgRNA7 (Fig. 1). When a large deletion has occurred smaller PCR products than the expected ones from wild-type (WT) controls will be visible on an agarose gel. Additionally, the sgRNA6 target site plus PAM contains the recognition sequence for restriction enzyme *Xcm*I (CCANNNNN↓NNNNTGG). When a small indel has occurred within this target site the PCR product will not be digested by *Xcm*I.

The PCR with primers Fw519 + Rv1925 flanking sgRNA6 (Fig. 1) did not yield PCR products obviously smaller than the 1407-bp WT PCR product in any of the primary transformants, indicating no large deletions had occurred in this region. In contrast, PCR with primers Fw2969 + Rv4230 flanking the three other sgRNAs resulted in the selection of 17 T1 transformants with large deletions compared to the 1262-bp PCR product of the WT allele. These were subsequently selfed to produce T2 progeny. T2 progenies were analyzed by PCR and individual T2 plants homozygous for the mutation were selected to produce T3 progenies to facilitate further characterization of the mutations (results shown in the next section).

To analyze whether any of the selected T2 or T3 mutants contained a small indel at the sgRNA6 target site PCR products obtained with primers Fw519 + Rv1925 were digested with *Xcm*I. For all tested plants digestion was complete, suggesting that no mutation had occurred at the sgRNA6 target site. To be sure none of the mutants contained a mutation in the sgRNA6 region the PCR products were sequenced. No differences with the WT allele were observed. These results indicate that sgRNA6 in the FKS1dom1 domain was not effective in producing mutations, whereas one or more of the three sgRNAs in the Glucan-synthase domain were successful.

### Characterization of CRISPR/Cas-9-mediated mutants in *SlPMR4*

**Fig. 2:**
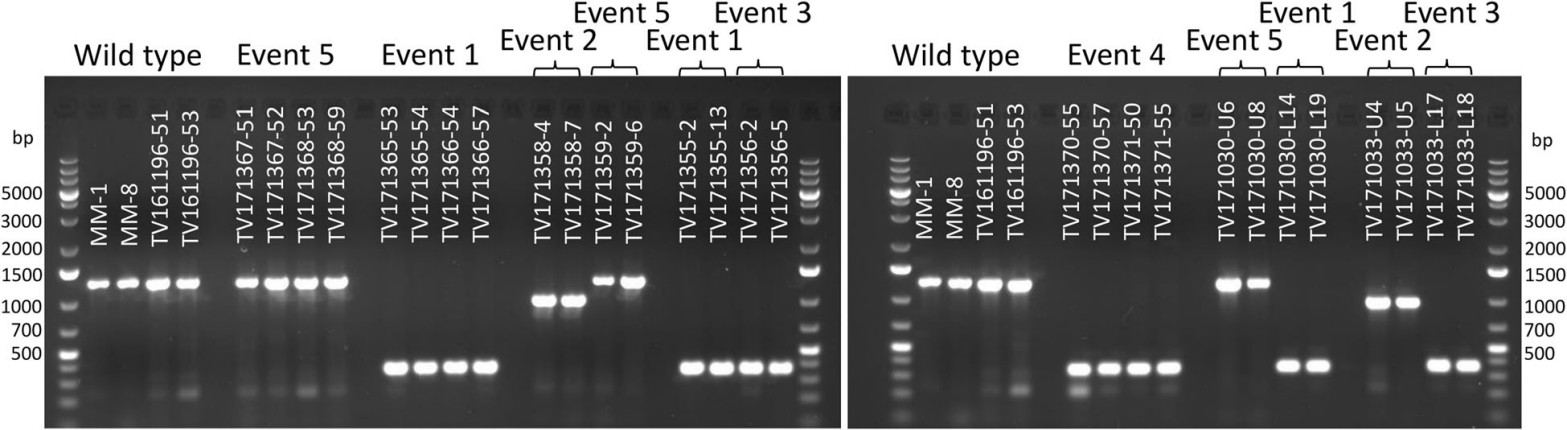
PCR amplification of the CRISPR tomato mutants. Selection of the mutants was done by amplifying the region containing sgRNAs 8, 1 and 7 using flanking primers Fw2969 and Rv4230 (Fig. 1). A 1262-bp wild-type allele was amplified in Moneymaker (MM) and transformant TV161196 (non-mutant). Smaller fragments than the wild-type allele indicate deletions between sgRNAs. Different mutation events are indicated and sequence details of events 1 to 5 are given in Fig. 3

We characterized the mutation events in T2 and T3 progenies derived from the original transgenic T1 plants (Table 1). From segregating T2 families plants homozygous for potential mutant alleles were selected that showed size differences in the PCR amplified products (using primers Fw2969 + Rv4230) when compared to the WT allele. In addition, homozygous T3 lines were obtained and the presence of mutant alleles was verified by repeating the PCR amplification (Fig. 2).

**Table 1:** Overview of the mutation events in the tomato *slpmr4* CRISPR mutants

Event	Mutation	T2 plants	T3 lines
1	900-bp deletion	TV171009-L TV171030-L	TV171365 TV171366 TV171355
2	5-bp deletion and 277-bp deletion	TV161212-U TV171033-U	TV171358
3	895-bp deletion	TV161212-L TV171033-L	TV171356
4	902-bp deletion and T insertion	TV171010	TV171370 TV171371
5	4-bp deletion and 892-bp inversion	TV171009-U TV171030-U	TV171367 TV171368 TV171359

For each mutation event the mutation is described and the homozygous plants (T2) and homozygous lines (T3) are listed

*L* Lower band of PCR products in heterozygous plants, *U* Upper band of PCR products in heterozygous plants

**Fig. 3:**
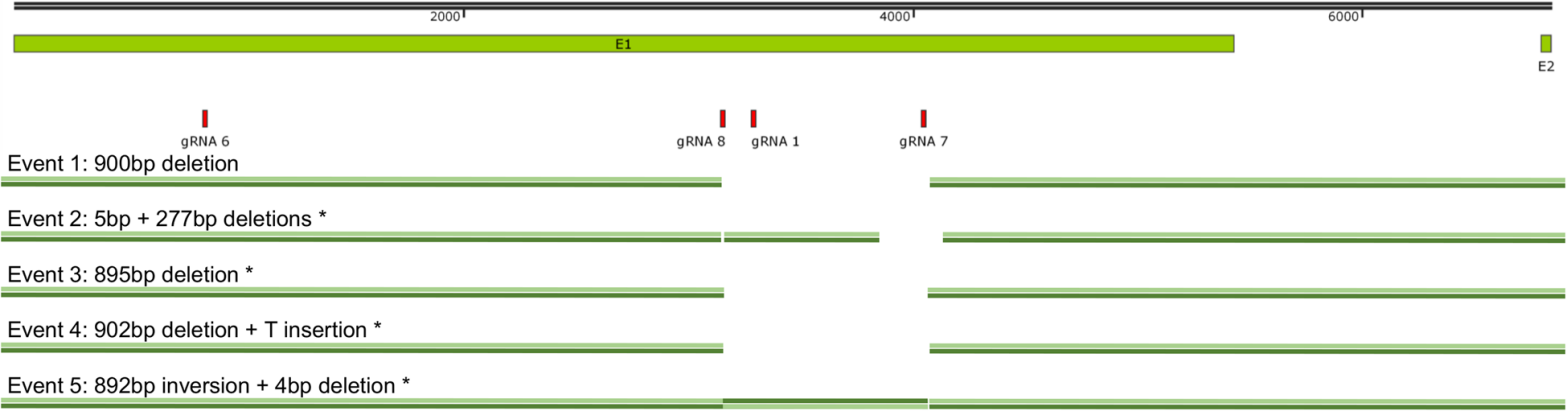
Schematic representation of the mutation events in *SlPMR4* mutants. Two exons (E1 and E2) of the *SlPMR4* gene are shown. The positions of the four sgRNAs are shown in red. The mutation for each event is represented at the genomic level. Deletions ranging from 4-bp to 902-bp were observed. Additionally, a 892-bp inversion is present in the event 5 mutants. * indicates the presence of premature translation termination codons (PTTCs) in the predicted protein

By sequencing of PCR fragments we identified five different mutation events (“events”) in the mutant lines (Fig. 3, Table 1 and Supplementary Document 1). Event 1 contains a 900-bp in-frame deletion between sgRNA8 and sgRNA7. Event 2 shows deletions of 5 bp at sgRNA8 and 277 bp at sgRNA7. Event 3 carries a 895-bp deletion between sgRNA8 and sgRNA7. Event 4 has a 902-bp deletion between sgRNA8 and sgRNA7 and the insertion of a T at the site of the mutation. Event 5 is a special case that has a 4-bp deletion and a 892-bp inversion next to the deletion between sgRNA8 and sgRNA7. With the exception of event 1, all the mutation events are predicted to generate premature translation termination codons (PTTCs) in the transcript (Supplementary figure1). The predicted protein of event 1 is lacking 300 amino acids in the Glucan-synthase domain.

Phenotypically, the CRISPR/Cas9 *pmr4* mutants were similar to non-transformed MM. However, they displayed a slight reduction in size when compared to MM and WT allele-carrying transformants. An exception to this is line TV171370 that showed segregation of plants with a dwarf phenotype (three out of eight T3 progeny). To investigate the cause of this dwarf phenotype a PCR was performed to check for the presence of the Cas9 sequence and possible association with T-DNA integration site. All eight plants carried the Cas9 gene; therefore no association was found with T-DNA integration. Next, the sgRNA6 target site was investigated by sequencing of the Fw519 + Rv1925 PCR product. No additional mutation(s) were observed in the dwarf plants.

One of the criteria for selection of the sgRNAs was a minimal chance of off-targets (unintended mutations elsewhere in the genome instead of *SlPMR4*). When using the Cas-OFFinder program [34] with a threshold of 3 or less mismatches between sgRNA and (off-) target sequence no putative off-targets were predicted for sgRNA 6, one for sgRNA8 (in an intergenic region), one for sgRNA1 (in an intron of gene Solyc09g009800) and three for sgRNA7 (Supplementary Table 2). These off-targets all contain 3 mismatches, most of which are present in the seed of the sgRNA. From the three off-targets of sgRNA7 two are in intergenic regions and one in the coding sequence of gene Solyc02g078230. This gene encodes a callose synthase-like gene designated *SlPMR4*-h2 [21]. Because of this we investigated whether any mutation had occurred in the *SlPMR4*-h2 gene in our selected *SlPMR4*(−h1) mutants. For this, a PCR was performed using primers PMR4_h2_Fw1 and PMR4_h2_Rv1 yielding a 765-bp PCR product containing the putative off-target site of sgRNA7. In total, PCR products of 36 individual T2 and T3 mutant plants and 6 control plants (MM and WT-allele carrying transgenic plants) were sequenced. No sequence differences were found between control and mutant (including the three dwarf) plants, indicating no off-target mutation had occurred in the *SlPMR4*-h2 gene.

### Resistance to powdery mildew in *slpmr4* mutants

**Fig. 4:**
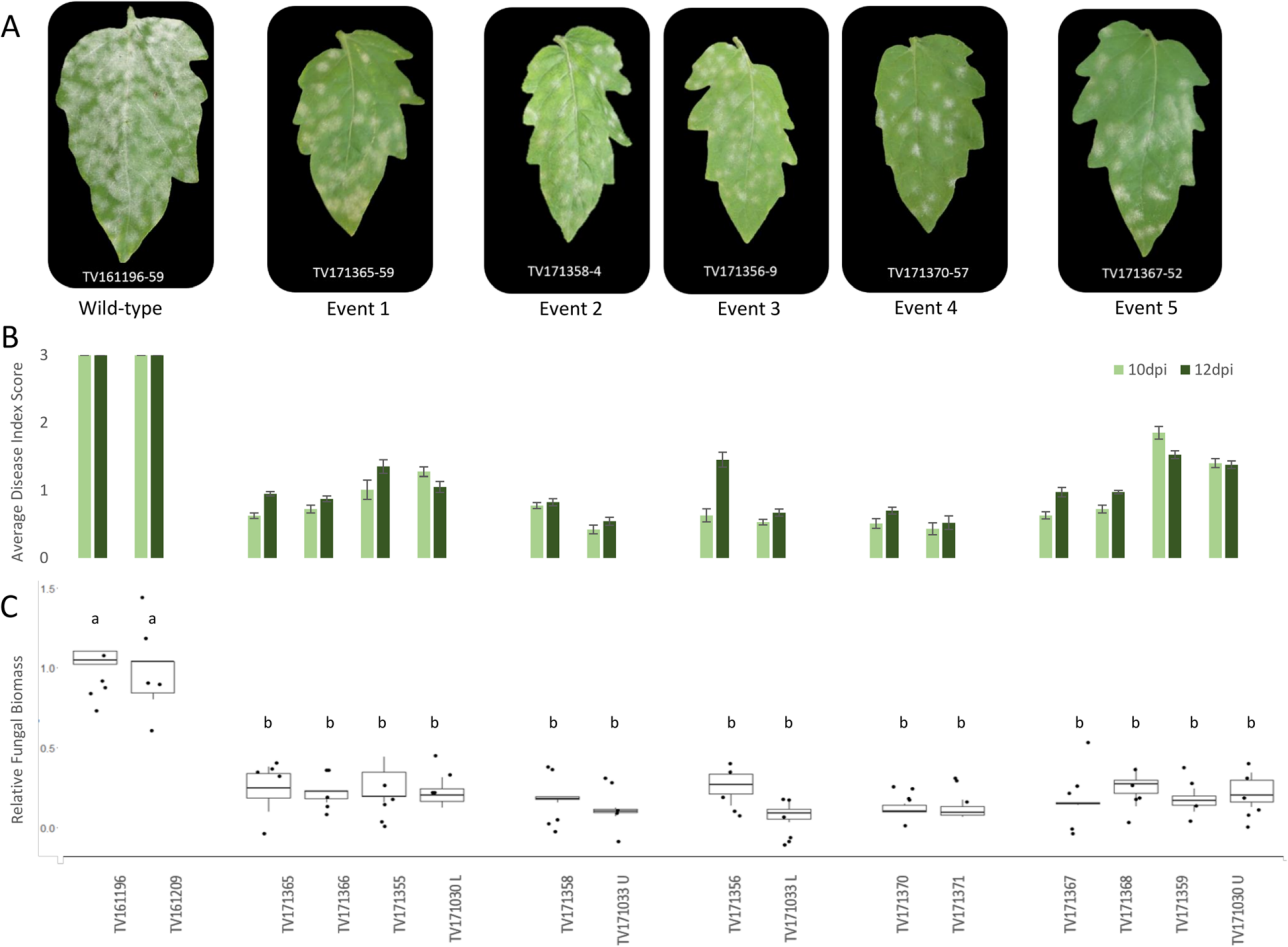
Phenotypic response of the *pmr4* mutants to infection with *Oidium neolycopersici*. **a**. Powdery mildew symptoms observed on the leaves of wild-type plants and mutants (one genotype is given from each of the five mutation events). Photos were taken at 21 days post inoculation (dpi). **b**. Average disease index score of the mutant lines at 10 and 12 dpi. **c**. Relative fungal biomass quantification on at least three individual plants of the mutant lines. This is calculated as the ratio of fungal ITS gene amplification in comparison with tomato EF1a and normalized with the values of the wild-type genotype TV161196. Significant differences were observed between the different mutation events and the control lines (Tukey HSD test, α = 0.05)

A previous study with RNAi lines showed that the knock-down of *SlPMR4* enhances resistance against powdery mildew [21]. To evaluate whether our *slpmr4* mutant lines showed increased or full resistance against PM, we inoculated them with *On* to assess the disease index (Fig. 4a and b). Additionally, we quantified the disease severity by measuring the relative *On* biomass in the mutants to confirm the phenotypic observations (Fig. 4c). Two unsuccessful transgenic lines (TV161196 and TV161209) carrying the WT allele were used as controls. No significant differences in the disease index or the relative fungal biomass were observed among the mutants. However, all the mutants displayed reduced susceptibility compared to the controls as indicated by a lower disease index and significantly lower fungal biomass (Fig. 4, Supplementary figure2).

**Fig. 5:**
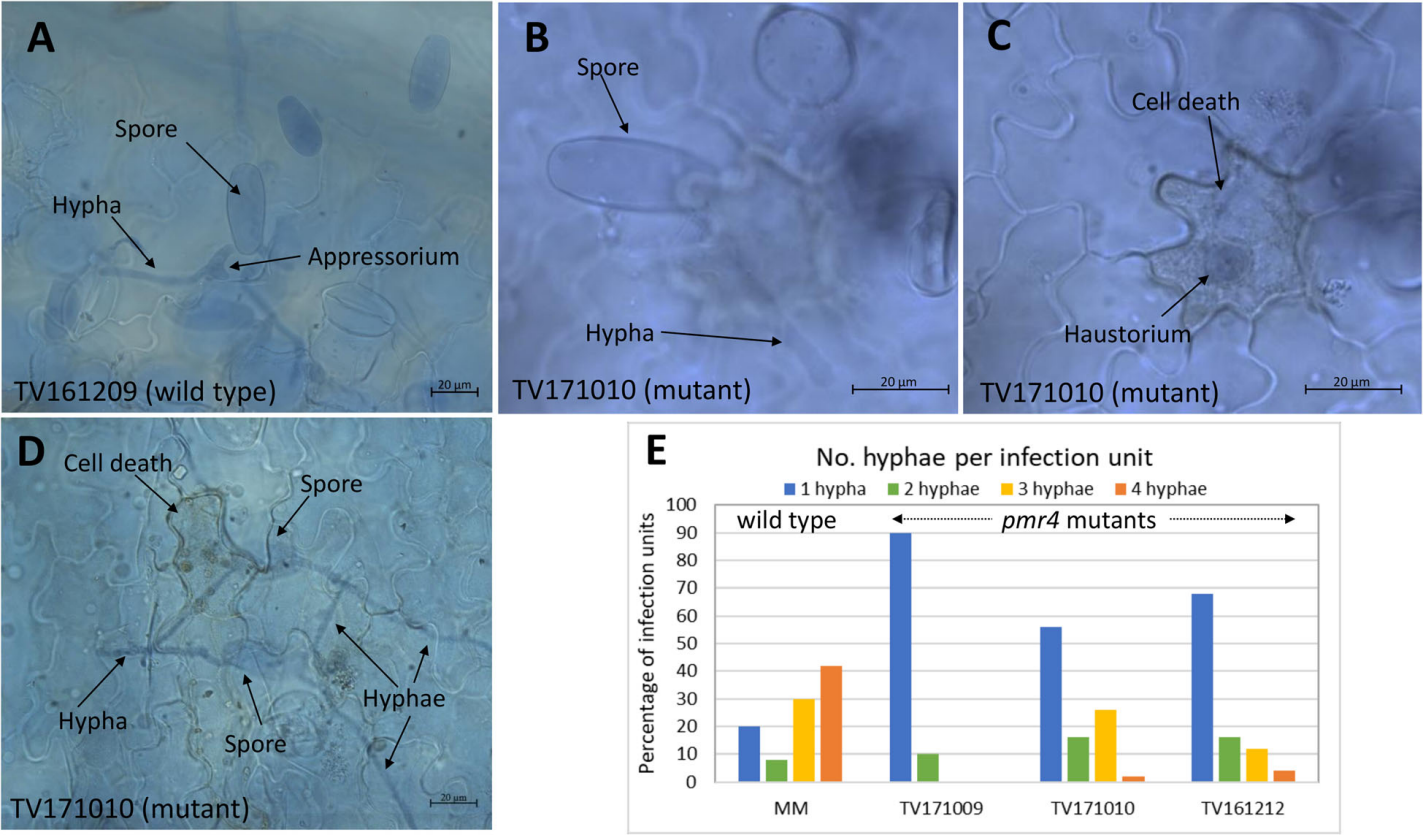
Microscopic observations on powdery mildew infection at 44 h post inoculation (hpi). **a.** In the wild-type allele-carrying plant (TV161209) a normal development of the spores occurs; appressorium and hyphae are developed. **b,c.** In the mutant plant (TV171010; event 4) cell death is observed in epidermal cells invaded by the fungus. **c** shows a deeper focal plane involving the same infection unit as in **b;** a haustorium is present in the cell showing cell death. **d.** Both cell death and hyphal growth in mutant plant TV171010. **e.** Comparison of number of hyphae per infection unit between wild type and *pmr4* mutant plants

Histological analysis of the infection sites of Arabidopsis *pmr4* mutants revealed the presence of hypersensitive response (HR)-like cell death [14]. To investigate whether this phenomenon could also be observed in our *slpmr4* mutants, we performed histological studies using heavily infected leaf samples of 21 plants representing all five mutation events in addition to wild-type MM plants. The samples were taken at 44 h post infection (hpi) and stained with trypan blue. Fungal structures and plant cell death were quantified in both mutant and wild-type plants (Table 2). No papillae were observed in any of the samples. HR-like cell death was visible at a much higher percentage of the infection sites in all *pmr4* mutants compared to the wild-type allele-carrying MM (Table 2 and Fig. 5). Simultaneously, the percentage of primary haustoria and the number of hyphae per infection unit was decreased in the mutants compared to MM. These results corroborate the finding that tomato *pmr4* mutants show reduced susceptibility to *On*, but not complete resistance.

**Table 2:** Histological study of *Oidium neolycopersici* growth

Genotype	*SlPMR4* mutation	Mutation event	Primary AP per IU (%)	Per AP		# Hyphae per IU (%)			
				Primary HS (%)	Primary HR (%)	1	2	3	4
MM	wild type	none	100	80	22	20	8	30	42
TV171009	bi-allelic	1 + 5	88	11.3^*^	81.8	90	10	0	0
TV171010	homozygous	4	96	47.9	91.6^*^	56	16	26	2
TV161212	bi-allelic	2 + 3	92	36.9	84.7^*^	68	16	12	4

Development of *Oidium neolycopersici* on the susceptible genotype Moneymaker (MM) and three *slpmr4* CRISPR mutant lines carrying different mutation events

Asterisks represent statistically significant differences between the mutant genotypes and cultivar MM as calculated by a t-test. *: *p* < 0.05

*IU* Infection unit, *AP* Appressorium, *HS* Haustorium, *HR* Hypersensitive response-like cell death

**Fig. 6:**
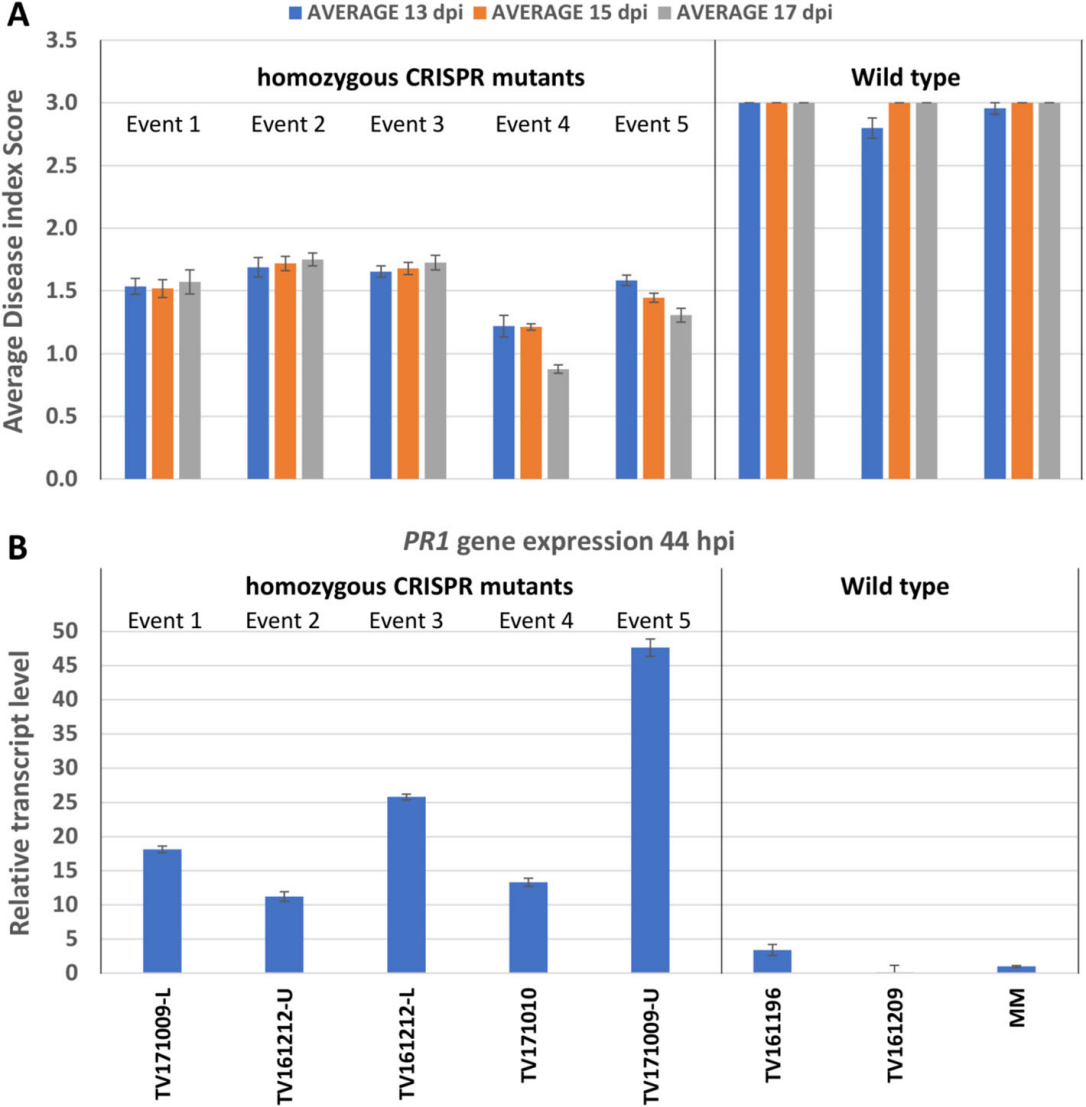
PM-resistance level and *PR1* gene expression of *slpmr4* mutant lines. **a.** Average disease index score of mutant and control lines at 13, 15 and 17 days post inoculation (dpi). **b.** Relative *PR1* gene expression in leaf samples of mutant and control lines 44 h post inoculation (hpi)

In Arabidopsis it was shown that PM resistance in the *pmr4* mutant is associated with an activation of the SA signal transduction pathway [14]. To analyze whether the SA pathway is also activated in our *slpmr4* mutants expression level of the tomato *PR1* gene 44 h after PM infection was determined by qPCR. Infected leaf samples were taken from individual homozygous mutant T2 plants representing all five mutation events and control plants (MM and unsuccessful transgenic lines TV161196 and TV161209). Disease index scores of this experiment are shown in Fig. 6a and relative *PR1* gene expression in Fig. 6b. Plants from all five mutation events show reduced susceptibility (but not complete resistance) to PM compared to the control lines (Fig. 6a). All mutant plants showed a significant increase in *PR1* gene expression at 44 h post infection (hpi) compared with the non-mutant controls (Fig. 6b).

## Discussion

### Effectiveness of multiple guide RNAs to obtain knock-out mutants

The use of loss-of-function alleles of *S*-genes in plant breeding is a promising alternative to the traditional *R*-gene-based introgression breeding because of its durable and broad-spectrum characteristics [10]. In this study, we successfully produced CRISPR/Cas-9-mediated knock-outs of the susceptibility gene *PMR4* in tomato against the PM pathogen *On*. Our results showed that the use of four sgRNAs for CRISPR-induced mutation and selection using PCR amplification to screen for visible (large) deletions was efficient to obtain the five described mutation events. Analysis of the different target sites indicated a difference in effectiveness of the four sgRNAs although all four sgRNAs were selected based on the same stringent criteria such as GC content, secondary structure and base pairing scores (Supplementary Table 1). No mutations were found close to the sgRNA6 target site, thus it was not efficient in guiding the Cas9 protein to induce double-stranded breaks. However, all five described mutation events seem to be the result of double-stranded breaks at target sites of both sgRNA8 and sgRNA7, as deletions were found at or between these positions. Therefore, sgRNA8 and sgRNA7 seem to be highly effective. We cannot judge whether sgRNA1 was effective, as the target site of this sgRNA is positioned between those of sgRNA8 and sgRNA7, and was deleted in three of the five mutation events (Fig. 3). As we focused on the selection of mutants with large deletions in the *SlPMR4* gene we have not checked whether any additional mutants with small indels (insertion or deletions of a few nucleotides) at the targets sites of sgRNA1, 7 or 8 were present among the original primary transformants. Such an analysis would allow a better comparison of the effectiveness of sgRNAs1, 7 and 8.

One of the five mutation events we characterized contained a 892-bp inversion, showing that chromosome re-arrangement can occur by using CRISPR/Cas9 technology. In agreement with our results, genome editing via the CRISPR/Cas9 system has recently been reported to produce inversions between sgRNAs [35, 36]. Induction of inversions are of particular interest in plant breeding due to its potential to allow the fixing or breaking of linkages [36].

### Putative pleiotropic effects of *SlPMR4* mutation

Similar to the observation on transgenic plants in which *SlPMR4* (Solyc07g053980) was silenced by RNAi [21], a slight reduction in plant size was observed in the CRISPR/Cas9 *slpmr4* mutants. This may be due to an elevated SA level in the mutants. Constitutively high levels of SA can result in growth impairment in Arabidopsis [37, 38]. Although we did not measure SA levels in our mutant lines, expression of the *PR1* gene (as an indicator of SA levels) was increased in PM-infected leaves of the *slpmr4* mutants compared to controls containing WT alleles of *SlPMR4*.

Three out of eight individuals of T2 family TV171370 showed a dwarf phenotype. We tried to identify the cause for this dwarf phenotype. None of the plants showed additional mutations at the sgRNA6 target site of *SlPMR4* or at the possible off-target site in Solyc02g078230 (*SlPMR4-*h2). Furthermore, all eight plants were positive for the Cas9 gene. Therefore, no obvious cause for the occurrence of dwarf plants was identified. However, it is still possible that the site of T-DNA integration plays a role. When the T-DNA of the CRISPR/Cas9 construct has integrated within a gene influencing plant growth, dwarf plants are obtained only when the T-DNA insertion is present in homozygous state. Another possibility is that multiple integrations of the T-DNA had occurred in the parental T1 plant of TV171370, one of which would be in a crucial gene for plant development.

As dwarf plants were only found in one T2 family and not in any of the others this phenotype does not seem to be caused by mutation of the *SlPMR4* gene. The possible pleiotropic effects in loss-of-susceptibility lines have been discussed as a setback to the deployment of *S*-genes in plant breeding [9]. Clearly, further phenotypic analyses in standard greenhouse conditions and yield estimation should determine whether the gain in resistance of *pmr4* tomato plants also carries a fitness cost in the plants.

### Resistance to PM in knock-out *SlPMR4* plants

We aimed to investigate whether the full knock-out of *SlPMR4* would result in a higher level of resistance than RNAi-silenced transgenic plants [21] . In this study we have shown that our *slpmr4* mutants in the susceptible cultivar Moneymaker background displayed enhanced resistance to tomato PM compared to control plants containing WT alleles of *SlPMR4*, but not complete resistance. We evaluated the plants for disease index at 10–17 dpi (Figs. 4 and 6) and determined fungal biomass at 21 dpi. It could be argued that susceptibility in the mutants is not reduced but delayed. However, we kept the inoculated *pmr4* mutants till 30–35 dpi and still the plants showed less infection than the WT control plants. Therefore, the *pmr4* mutants show reduced susceptibility to PM.

In agreement with previous observations in Arabidopsis *pmr4* mutants [14] we observed a higher occurrence of HR-like cell death at infection sites in tomato CRISPR *pmr4* mutants compared to wild type plants. This increased HR likely resulted from activation of the SA signaling pathway, as *PR1* gene expression was significantly increased in the *pmr4* mutants compared to the controls. Still, this cell death did not completely block fungal growth in the tomato *pmr4* mutants. In barley, RNAi was used to downregulate gene *HvGsl6*, which is the closest ortholog of *AtGSL5* (*PMR4*) [39]. As expected, this resulted in a lower accumulation of papillary and wound callose. However, contrary to what has been found in Arabidopsis and tomato, silencing of *HvGsl6* led to a higher susceptibility of barley to PM *Blumeria graminis* f. sp. *hordei* compared with control lines. It was found that silencing of *HvGsl6* does not lead to activation of the SA-dependent defense pathway. Whether reduced susceptibility to powdery mildew species in *pmr4*/*gsl5* mutants is a plant species-specific phenomenon remains to be investigated. We have not been able to check for presence of callose at the cell wall near the sites of fungal penetration in the CRISPR mutants, although we would expect absence or a lower level of callose compared to wild-type plants, depending on redundancy of callose synthase genes. In order to verify whether the PM resistance observed in the tomato *pmr4* mutants is callose-independent, quantification of callose deposition should be included in future experiments with these mutants after PM attack.

In tomato an additional possible ortholog of *PMR4*, designated *SlPMR4*-h2 (Solyc02g078230), has been found [21]. This gene is the closest tomato ortholog to Arabidopsis gene At4g04970 [21], also known as *GSL1*/*CalS11*. Both Solyc07g053980 (*GSL5*/*CalS12*-like) and Solyc02g078230 (*GSL1*/*CalS11*-like) are reported to function in callose formation during pathogen infection [40]. It would be interesting to investigate whether these genes show functional redundancy in relation to PM resistance/susceptibility, and thus whether knocking out both genes simultaneously would result in higher resistance to PM. However, GSL1 and GSL5 also play redundant roles in plant development [15] . In Arabidopsis, a *gsl5* mutant with one mutant allele of *GSL1* is severely stunted and shows highly reduced fertility, and double mutants could not be obtained [15].

### Perspectives for *slpmr4* mutants in breeding for resistance against PM

We have transformed susceptible tomato cultivar Moneymaker with the *SlPMR4* CRISPR construct and observed reduced susceptibility in the obtained mutants associated with the activation of the SA signaling pathway. It would be interesting to know what effect the *slpmr4* mutation would have in different genetic backgrounds, and especially in resistant tomato backgrounds, with the aim to pyramid different resistance genes. As resistance conferred by the *pmr4* mutation is associated with an elevated SA pathway defense response we expect that PM resistance in tomato lines carrying *Ol-4* or *Ol-6* would not be influenced by mutation of the *SlPMR4* gene. *Ol-4* and *Ol-6* encode NB-LRR type resistance genes [41] and lines containing these genes show a fast HR after PM infection. This strong resistance is not expected to become even stronger because of further elevation of SA levels caused by the *pmr4* mutation. Resistance genes *Ol-1*, *Ol-3* and *Ol-5* are not cloned yet, but lines containing these genes show a slow HR response. Possibly, combining one of these genes with the *pmr4* mutation might result in a stronger or faster HR response. PM resistance conferred by the recessive gene *ol-2*, containing a mutation in the *SlMlo1* gene, is associated with formation of papillae and increased callose deposition. It would be interesting to analyze the PM resistance level in double mutants *slmlo1 slpmr4*. The resistance conferred by *ol-2* might be compromised when the callose synthase gene *SlPMR4* is mutated and there is no redundancy with another callose synthase gene (e.g. *SlPMR4*-h2). However, in Arabidopsis the *atmlo2 atpmr4* double mutant displayed the same level of resistance to PM *G. orontii* as the single *atmlo2* mutant [42]. Therefore, the *atmlo2* resistance was independent of PMR4-mediated callose deposition [42]. Whether the same holds true in tomato and the *slmlo1 slpmr4* double mutant would still show the same level of resistance as the single *slmlo1* (*ol-2*) mutant remains to be tested.

## Conclusions

The use of *S*-genes in plant breeding stands as a promising alternative due to its durable and broad-spectrum characteristics. In this study, we used CRISPR/Cas-9 targeted mutagenesis to knock-out the *S*-gene *PMR4* in tomato. We characterized five different mutation events and confirmed the reduced susceptibility of the mutant lines against *On.* Our study demonstrates the efficiency and versatility of the CRISPR/Cas9 system as a powerful tool to study and characterize *S*-genes.

## Methods

### Design of gRNAs and transformation

The full-length CDS of the tomato *PMR4* homolog (Solyc07g053980) was retrieved from the Sol Genomics Network database [43]. Four single guide RNAs (sgRNAs) targeting the gene were selected using the guidelines described by Liang et al. [35] (Supplementary Table 1). A first list of gRNAs was generated using the CC-Top CRISPR/Cas9 Target Prediction Tool [44]. The G + C content of the sgRNAs was calculated using the ENDMEMO webtool (http://www.endmemo.com/bio/gc.php). The folding of the gRNAs was predicted using the Mfold web server [45]. Additionally, the activity of the gRNAs was predicted using the sgRNA scorer [46]. Four sgRNAs were selected (sgRNA1:TTAAAGCAGTCCCATACTCG, sgRNA6: GTACTGCCCCACACTCTGCG, sgRNA7: GCCAAGGTTGCCAGTGGCAA, and sgRNA8: GGATATCAGAGAAGGATCAG) for transformation. The analysis of the location of the sgRNAs and topology of the predicted protein was made using a set of twelve plasmids obtained from Addgene was used to build the construct used for transformation: pICH86966 (as template for amplification); pICSL01009 (as level 0 plasmid); pICH47751, pICH47761, pICH47772, pICH47781 and pICH47732 (as level 1 plasmids); pICH41766, pICH41780 and pICH41822 (as linkers); and pAGM4723 (as level 2 binary vector) (Supplementary figure3). The plasmids were cloned using *E. coli* DH5α and transformed to Agrobacterium strain AGL1. Susceptible tomato cultivar Moneymaker (from WUR-Plant Breeding seed collection) was used for transformation according to the method described in [47] according to Dutch legislation under GMO licence 01–135.

### PCR-based selection of *slpmr4* mutants and characterization of mutation events

Selection of plants carrying deletions in *SlPMR4* was done by analyses of PCR products flanking the sgRNA target regions. PCR using primers Fw519 (5′- TGGTGCTCTTTTCTCGGTCT-3′) and Rv1925 (5′-CAACTGCTCTTCTGGCATCA-3′) yields a 1407-bp product flanking the sgRNA6 target for the WT allele, and PCR with primers Fw2969 (5′-GCGAATGCGTAGAGAAGGAA-3′) and Rv4230 (5′-CCCCACTAAGTGCCAGGTAA-3′) yields a 1262-bp PCR product flanking sgRNAs8, 1 and 7 for the WT allele. Smaller sizes of the amplified fragments in transgenic plants compared to the WT allele indicated deletions in the targeted region. The sgRNA6 target site together with the PAM site contains the recognition sequence for restriction enzyme *Xcm*I. Fw519 + Rv1925 PCR products were digested with this enzyme (New England Biolabs) yielding 1069-bp and 338-bp fragments for the WT *SlPMR4* allele. The PCR products were sequenced to further characterize the mutation events. Primary transformants (T1) carrying mutant alleles were selected using these methods and selfed to produce T2 progeny. Homozygous plants from two T2 bi-allelic lines (TV171030 and TV171033) were selected for use in the disease assay. Homozygous mutant T2 plants derived from other mono-or bi-allelic T1 transformants were selected and selfed to obtain T3 progeny. These included TV161212-U, TV161212-L, TV171009-U, TV171009-L and TV171010 (where U stands for upper band of PCR products in the agarose gel, and L for lower band). Plants from the individual T2 plants and T3 mutant lines (TV171367, TV171368, TV171359, TV171365, TV171366, TV171355, TV171358, TV171356, TV171370 and TV171371) derived from the selfing of previously selected T2 plants, were also tested in the disease assay.

### Off-target analysis

The program Cas-OFFinder [34] at http://www.rgenome.net/cas-offinder/ was used to check for possible off-targets of the four sgRNAs of *SlPMR4*. Mismatch number was set at 3 or less. To analyze possible off-target mutations in gene Solyc02g078230 (*SlPMR4*-h2) a PCR was performed using primers PMR4_h2_Fw1 (5′-AACGTGTTCTTGCCGATCCTC-3′) and PMR4_h2_Rv1 (5′-CAAAGTGGCTGCGAGCATACA-3′), yielding a 765-bp PCR product for the WT *SlPMR4*-h2 allele. PCR products of *slpmr4* mutants were sequenced and compared with WT control sequences.

### Disease assay and quantification of relative fungal biomass

Ten plants homozygous for each of the alleles in the T2 lines and ten plants from the homozygous T3 lines were inoculated with the Wageningen University isolate of *On* by spraying 4 weeks-old plants with a suspension of conidiospores obtained from leaves of infected tomato Moneymaker plants and adjusted to a concentration of 3.5*10^4^ spores per ml. Two transgenic lines (TV161196 and TV161209) obtained from the same CRISPR transformation experiment, but carrying the wild-type allele were used as controls. Inoculated plants were grown at 20 ± 2 °C with 70 ± 15% relative humidity and day length of 16 h in a greenhouse of Unifarm of Wageningen University & Research, The Netherlands. Disease index scoring was carried out 10 and 12 days after inoculation. Powdery mildew symptoms were scored visually using a scale from 0 to 3 as described by Bai et al. [7]. For the quantification of relative fungal biomass, plant and fungal genomic DNA was isolated from infected leaves collected at 21 dpi, using an adapted CTAB protocol [48]. 10 ng of DNA were used as a template for amplification. Relative fungal biomass was quantified by real-time PCR using the primer pairs On-Fw/On-Rev amplifying the internal transcribed spacer sequence (ITS) of *Oidium neolycopersici* [49] and Ef-Fw (5′-GGAACTTGAGAAGGAGCCTAAG-3′)/Ef-Rev (5′-CAACACCAACAGCAACAGTCT-3′) amplifying tomato reference gene *Elongation Factor 1α* (*Ef1α*) [50]. The 2^-ΔΔCt^ method [51, 52] was used to calculate the fold-change of the ratio between fungal and tomato gDNA.

### Histological analysis

At least two plants of each line were grown together with the plants in the disease assay described above but were infected using a heavier inoculation of 3*10^5^ spores per ml. Infected leaf samples of 4 cm^2^ were collected 44 h post inoculation, bleached in a 1:3 (v/v) acetic acid/ethanol solution, and stained 48 h later by boiling in 0.005% trypan blue in lactophenol: ethanol (1:2 v/v) solution for 3–5 min and cleared in a nearly saturated aqueous solution of chloral hydrate (5:2 w/v) as described by [53]. The slides were mounted on glass slides with a 1:1 (v/v) glycerol-water solution. Analysis of the slides was done using a Zeiss Axiophot bright field microscope. For quantification of fungal structures and host cell death 50 infection units (*O. neolycopersici* conidia) were analyzed per genotype, from two slides obtained from two different plants per genotype.

### Analysis of *PR1* expression

Expression level of the tomato *PR1* gene was measured 44 hpi after infection with PM by qPCR. Infected leaf samples were taken from individual homozygous mutant T2 plants representing all five mutation events and control plants (MM and unsuccessful transgenic lines TV161196 and TV161209). Leaf samples were frozen in liquid nitrogen and stored at − 80 °C before being grinded into a fine powder using a pestle and mortar. Total RNA isolation was done using the MagMax™ 96 Total RNA Isolation Kit (Qiagen, Germany). cDNA synthesis was done using the iScript™ cDNA Synthesis Kit (Bio-Rad Laboratories, U.S.A.). 10 ng of cDNA were used as template for the reaction. Expression levels of *PR1* were measured using primers SlPR1a_Fw (5′-GTGTCCGAGAGGCCAGACTA-3′) and SlPR1a_Rev (5′-CATTGTTGCAACGAGCCC GA-3′), and compared to the expression of tomato *Ef1α* reference gene using primers Ef_Fw (5′-ATTGGAAACGGATATGCCCCT-3′) and Ef_Rev (5′-TCCTTACCTGAACGCCTGTCA-3′) [21].

## Supplementary information

**Additional file 1: Supplementary Document 1.** Alignment of sequences of PCR products of the tomato *PMR4* CRISPR mutant alleles. A. Sequence alignment of PCR products obtained by using primers Fw2969 and Rv4230 for wild type Moneymaker (MM) and five *PMR4* CRISPR mutation events. Primers and sgRNA1, 7 and 8 are indicated. In red, nucleotides differing form the MM allele are shown. Deletions are indicated by dashes. Event 5 contains a large inversion, indicated in red. **B.** Plot showing the inversion (blue line) of the sequence between sgRNA8 and sgRNA7 in mutation event 5 compared to MM.

**Additional file 2: Supplementary Figure 1.** Alignment of predicted proteins of the tomato *PMR4* CRISPR mutant alleles. Protein sequences are based on DNA sequencing data from the fragments amplified by the region flanked by primers Fw2969 and Rv4230.

**Additional file 3: Supplementary Figure 2.** Panel of phenotypes upon infection with ***Oidium neolycopersici.*** Leaves from wild-type allele-carrying controls and individual plants of the different *slpmr4* mutation classes are shown. Heavy fungal sporulation is present on the leaves of the wild-type plants, while less infection is seen on the leaves of the mutant plants.

**Additional file 4: Supplementary Figure 3.** Map of the level 2 vector for CRISPR/Cas9 transformation. The NPTII, Cas9, the four sgRNAs and AtU6 promoters are highlighted.

**Additional file 5: Supplementary Table 1.** Characteristics of four selected sgRNAs for *SlPMR4*. sgRNAs were selected using CCTop program. PAM, protospacer adjacent motif.

**Additional file 6: Supplementary Table 2.***SlPMR4* sgRNAs off-targets. Off-targets with a maximum of three mismatches were found for sgRNA8 and sgRNA7 with Cas-OFFinder [34]. crRNA, sgRNA sequences. DNA, off-target sequences. Mismatches are indicated in red.

## Data Availability

The sequences obtained by Sanger sequencing in this study are available in the GenBank database (https://www.ncbi.nlm.nih.gov/genbank/) under reference accession numbers MT521499 to MT521504. The data produced for this article are included within the manuscript and additional files, and the raw data are available from the corresponding author on reasonable request.

## Abbreviations

CRISPR: Clustered Regularly Interspaced Short Palindromic Repeats
Cas: CRISPR-associated systems
dpi: Days post inoculation
hpi: Hours post inoculation
NIL: Near-isogenic line
*On*: *Oidium neolycopersici*
PAM: Protospacer adjacent motif
PM: Powdery mildew
*PMR4*: Powdery Mildew Resistance 4
PTTC: Premature translation termination codon
*R* gene: *Resistance* gene
SA: Salicylic acid
sgRNA: Single-guide RNA
*S* gene: *Susceptibility* gene
WT: Wild type
