# Supplementary material for: CRISPR/Cas9-targeted mutagenesis of the tomato susceptibility gene PMR4 for resistance against powdery mildew
Source: BMC Plant Biol. 2020 Jun 19;20:284. doi: 10.1186/s12870-020-02497-y (PMC7304142; doi:10.1186/s12870-020-02497-y)

A Alignment of sequences of PCR product Fw2969+Rv4230 of MM and CRISPR PMR4 events

```
                 Fw2969→
MM               tagagaaggaatggttgatgagaagaaagagttatggactacaaagctaagggatcttcg
Event1           tagagaaggaatggttgatgagaagaaagagttatggactacaaagctaagggatcttcg
Event2           tagagaaggaatggttgatgagaagaaagagttatggactacaaagctaagggatcttcg
Event3           tagagaaggaatggttgatgagaagaaagagttatggactacaaagctaagggatcttcg
Event4           tagagaaggaatggttgatgagaagaaagagttatggactacaaagctaagggatcttcg
Event5           tagagaaggaatggttgatgagaagaaagagttatggactacaaagctaagggatcttcg

MM               tctttgggcatcatacagagggcagactcttactcgcacggttagggggatgatgtacta
Event1           tctttgggcatcatacagagggcagactcttactcgcacggttagggggatgatgtacta
Event2           tctttgggcatcatacagagggcagactcttactcgcacggttagggggatgatgtacta
Event3           tctttgggcatcatacagagggcagactcttactcgcacggttagggggatgatgtacta
Event4           tctttgggcatcatacagagggcagactcttactcgcacggttagggggatgatgtacta
Event5           tctttgggcatcatacagagggcagactcttactcgcacggttagggggatgatgtacta

                                                                 sgRNA8→
MM               ctatcgagctctcaaaatgctggcctttctggattctgcttgtgagatggatatcagaga
Event1           ctatcgagctctcaaaatgctggcctttctggattctgcttgtgagatggatatcagaga
Event2           ctatcgagctctcaaaatgctggcctttctggattctgcttgtgagatggatatcagag-
Event3           ctatcgagctctcaaaatgctggcctttctggattctgcttgtgagatggatatcagaga
Event4           ctatcgagctctcaaaatgctggcctttctggattctgcttgtgagatggatatcagat-
Event5           ctatcgagctctcaaaatgctggcctttctggattctgcttgtgagatggatatcagaga

                         PAM
MM               aggatcagtggaacttggttctatgaggcatgatgatagcattggtggtttaagttcaga
Event1           ------------------------------------------------------------
Event2           ----tcagtggaacttggttctatgaggcatgatgatagcattggtggtttaagttcaga
Event3           aggatca-----------------------------------------------------
Event4           ------------------------------------------------------------
Event5           aggattggcaaccttggcttcaaacatagatatctgattcaacccaacatcccttccttt


MM               aagatctcagtcttcgaggaggttgagtagagctgacagttcagtgagtatgttgtttaa
Event1           ------------------------------------------------------------
Event2           aagatctcagtcttcgaggaggttgagtagagctgacagttcagtgagtatgttgtttaa
Event3           ------------------------------------------------------------
Event4           ------------------------------------------------------------
Event5           gccaacttgtatatactcatggtgagtgacattgccacctcgtaatgtacagttgaagcc

                   PAM      ←sgRNA1
MM               aggccacgagtatgggactgctttaatgaaattcacatatgtggtagcttgtcagatata
Event1           ------------------------------------------------------------
Event2           aggccacgagtatgggactgctttaatgaaattcacatatgtggtagcttgtcagatata
Event3           ------------------------------------------------------------
Event4           ------------------------------------------------------------
Event5           agcaaaaatgtcctcgctgatgttgatcactttagatgccttgcttattcctcccctagt

MM               tggggctcagaaggccaaaaaagatccacatgcagaggaaattttgtatctgatgaaaaa
Event1           ------------------------------------------------------------
Event2           tggggctcagaaggccaaaaaagatccacatgcagaggaaattttgtatctgatgaaaaa
Event3           ------------------------------------------------------------
Event4           ------------------------------------------------------------
Event5           tagaaaccaaaacctgtcaaatacatctggatgtccataatgcattcggattttcagggg
```

```
MM              taatgaagctcttcgtgtagcttatgttgatgaggttcccacaggaagggatgagaagga
Event1          ------------------------------------------------------------
Event2          taatgaagctcttcgtgtagcttatgttgatgaggttcccacaggaagggatgagaagga
Event3          ------------------------------------------------------------
Event4          ------------------------------------------------------------
Event5          gttggctaatacacgctgtcctagggttacaaaactcatttcctgagctgacatgaacca

MM              ttattattctgtgcttgtgaagtatgatcaaaaacttgaaagggaagttgagatctatcg
Event1          ------------------------------------------------------------
Event2          ttattattctgtgcttgtgaagtatgatcaaaaacttgaaagggaagttgagatctatcg
Event3          ------------------------------------------------------------
Event4          ------------------------------------------------------------
Event5          agcaagggatgacacggaaccagtaaatatatgttctcgaactccaagaatcgtaggttt

MM              agttaagttgcctggtcctttgaagcttggggaggggaaaccagaaaatcaaaatcatgc
Event1          ------------------------------------------------------------
Event2          agttaagttgcctggtcctttgaagcttggggaggggaaaccagaaaatcaaaatcatgc
Event3          ------------------------------------------------------------
Event4          ------------------------------------------------------------
Event5          gcgaataccatagtagagtttgaattcttccaacaagttcctcattttcagtgcctcctc

MM              ctttatctttacccgtggtgatgcagttcagactattgacatgaaccaagataattactt
Event1          ------------------------------------------------------------
Event2          ctttatctttacccgtggtgatgcagttcagactattgacatgaaccaagataattactt
Event3          ------------------------------------------------------------
Event4          ------------------------------------------------------------
Event5          aaagtaattatcttggttcatgtcaatagtctgaactgcatcaccacgggtaaagataaa

MM              tgaggaggcactgaaaatgaggaacttgttggaagaattcaaactctactatggtattcg
Event1          ------------------------------------------------------------
Event2          tgaggaggcactgaaaatgaggaacttgttggaagaattcaaactctactatggtattcg
Event3          ------------------------------------------------------------
Event4          ------------------------------------------------------------
Event5          ggcatgattttgattttctggtttcccctccccaagcttcaaaggaccaggcaacttaac

MM              caaacctacgattcttggagttcgagaacatatatttactggttccgtgtcatcccttgc
Event1          ------------------------------------------------------------
Event2          caaacctacgattcttggagttcgagaacatatatttactggttccgtgtcatcccttgc
Event3          ------------------------------------------------------------
Event4          ------------------------------------------------------------
Event5          tcgatagatctcaacttccctttcaagtttttgatcatacttcacaagcacagaataata

MM              ttggttcatgtcagctcaggaaatgagttttgtaaccctaggacagcgtgtattagccaa
Event1          ------------------------------------------------------------
Event2          ttggttcatgtcagctcaggaaatgagttttgtaaccctaggacagcgtgtattagccaa
Event3          ------------------------------------------------------------
Event4          ------------------------------------------------------------
Event5          atccttctcatcccttcctgtgggaacctcatcaacataagctacacgaagagcttcatt
```

```
MM          ccccctgaaaatccgaatgcattatggacatccagatgtatttgacaggttttggtttct
Event1      ------------------------------------------------------------
Event2      ccccctgaaaatccgaatgcattatgga--------------------------------
Event3      ------------------------------------------------------------
Event4      ------------------------------------------------------------
Event5      attttcatcagatacaaaatttcctctgcatgtggatctttttggccttctgagcccc

MM          aactaggggaggaataagcaaggcatctaaagtgatcaacatcagcgaggacatttttgc
Event1      ------------------------------------------------------------
Event2      ------------------------------------------------------------
Event3      ------------------------------------------------------------
Event4      ------------------------------------------------------------
Event5      atatatctgacaagctaccacatatgtgaatttcattaaagcagtcccatactcgtggcc

MM          tggcttcaactgtacattacgaggtggcaatgtcactcaccatgagtatatacaagttgg
Event1      ------------------------------------------------------------
Event2      ------------------------------------------------------------
Event3      ------------------------------------------------------------
Event4      ------------------------------------------------------------
Event5      tttaaacaacatactcactgaactgtcagctctactcaacctcctcgaagactgagatct

                                                         sgRNA7→
MM          caaaggaagggatgttgggttgaatcagatatctatgtttgaagccaaggttgccagtgg
Event1      ------------------------------------------------------------
Event2      ------------------------------------------------------------
Event3      ------------------------------------------------------------
Event4      ------------------------------------------------------------
Event5      ttctgaacttaaaccaccaatgctatcatcatgcctcatagaa-ccaag-tt-ccactga

               PAM
MM          caatggagaacaagttcttagcagagatgtctataggttgggtcataggctggatttctt
Event1      caatggagaacaagttcttagcagagatgtctataggttgggtcataggctggatttctt
Event2      ------------------------------------------------------------
Event3      --atggagaacaagttcttagcagagatgtctataggttgggtcataggctggatttctt
Event4      caatggagaacaagttcttagcagagatgtctataggttgggtcataggctggatttctt
Event5      caatggagaacaagttcttagcagagatgtctataggttgggtcataggctggatttctt

MM          cagaatgctttctttcttttatacaactgtaggattcttcttcaatacaatgatgattgt
Event1      cagaatgctttctttcttttatacaactgtaggattcttcttcaatacaatgatgattgt
Event2      -----tgctttctttcttttatacaactgtaggattcttcttcaatacaatgatgattgt
Event3      cagaatgctttctttcttttatacaactgtaggattcttcttcaatacaatgatgattgt
Event4      cagaatgctttctttcttttatacaactgtaggattcttcttcaatacaatgatgattgt
Event5      cagaatgctttctttcttttatacaactgtaggattcttcttcaatacaatgatgattgt

                                                   ←Rv4230
MM          cctcactgtatatgcattcttatggggacgacttttacctggcacttagtgggg
Event1      cctcactgtatatgcattcttatggggacgacttttacctggcacttagtgggg
Event2      cctcactgtatatgcattcttatggggacgacttttacctggcacttagtgggg
Event3      cctcactgtatatgcattcttatggggacgacttttacctggcacttagtgggg
Event4      cctcactgtatatgcattcttatggggacgacttttacctggcacttagtgggg
Event5      cctcactgtatatgcattcttatggggacgacttttacctggcacttagtgggg
```

B Inversion of sequence between sgRNA8 and sgRNA7 in PMR4 CRISPR event 5

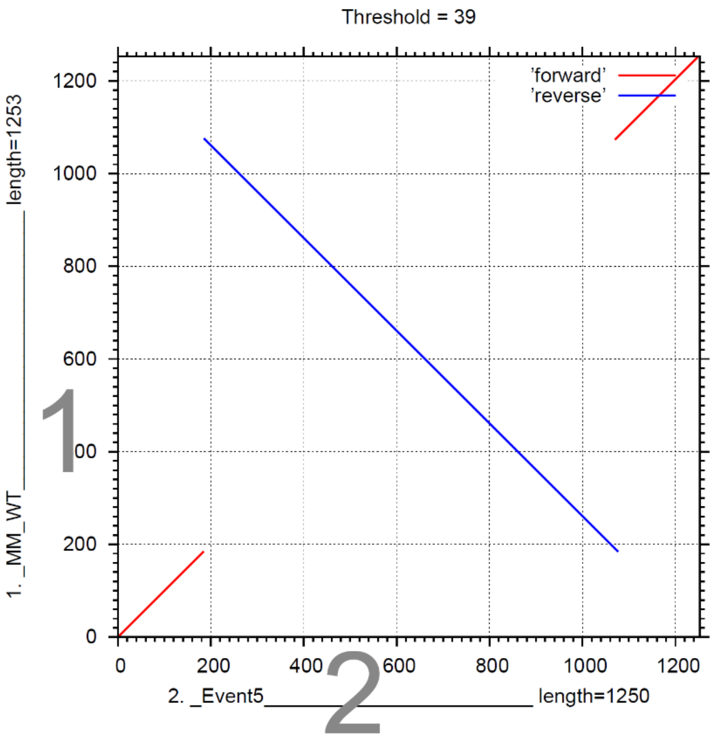

Supplement: Supplementary file 1 — Additional file 1: Supplementary Document 1. Alignment of sequences of PCR products of the tomato PMR4 CRISPR mutant alleles. A. Sequence alignment of PCR products obtained by using primers Fw2969 and Rv4230 for wild type Moneymaker (MM) and five PMR4 CRISPR mutation events. Primers and sgRNA1, 7 and 8 are indicated. In red, nucleotides differing form the MM allele are shown. Deletions are indicated by dashes. Event 5 contains a large inversion, indicated in red. B. Plot showing the inversion (blue line) of the sequence between sgRNA8 and sgRNA7 in mutation event 5 compared to MM. [file 12870_2020_2497_MOESM1_ESM.pdf]
