# Supplementary figures and images for: CRISPR/Cas9-targeted mutagenesis of the tomato susceptibility gene PMR4 for resistance against powdery mildew
Source: BMC Plant Biol. 2020 Jun 19;20:284. doi: 10.1186/s12870-020-02497-y (PMC7304142; doi:10.1186/s12870-020-02497-y)

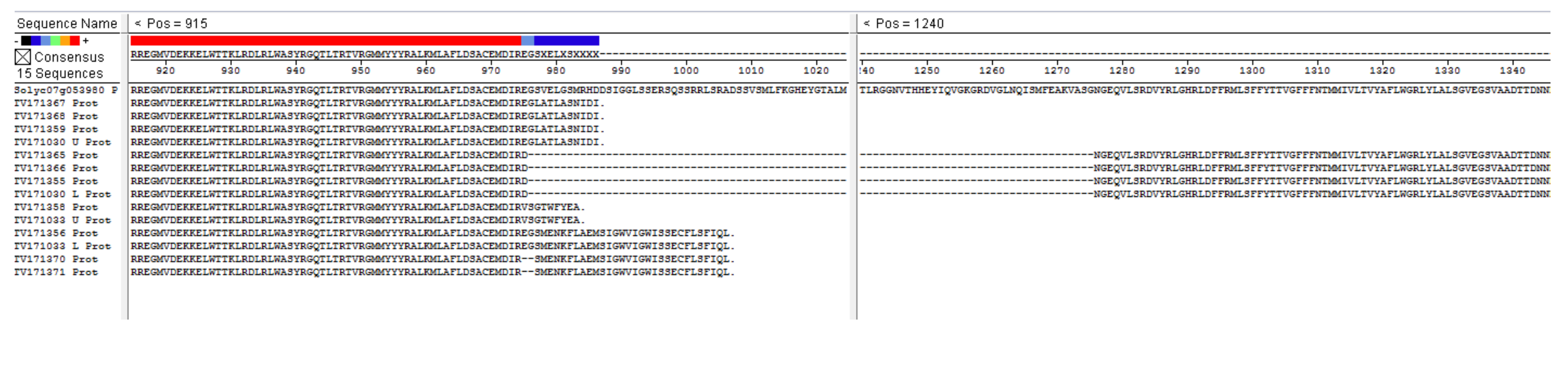

Supplement: Supplementary file 2 — Additional file 2: Supplementary Figure 1. Alignment of predicted proteins of the tomato PMR4 CRISPR mutant alleles. Protein sequences are based on DNA sequencing data from the fragments amplified by the region flanked by primers Fw2969 and Rv4230. [file 12870_2020_2497_MOESM2_ESM.tif]

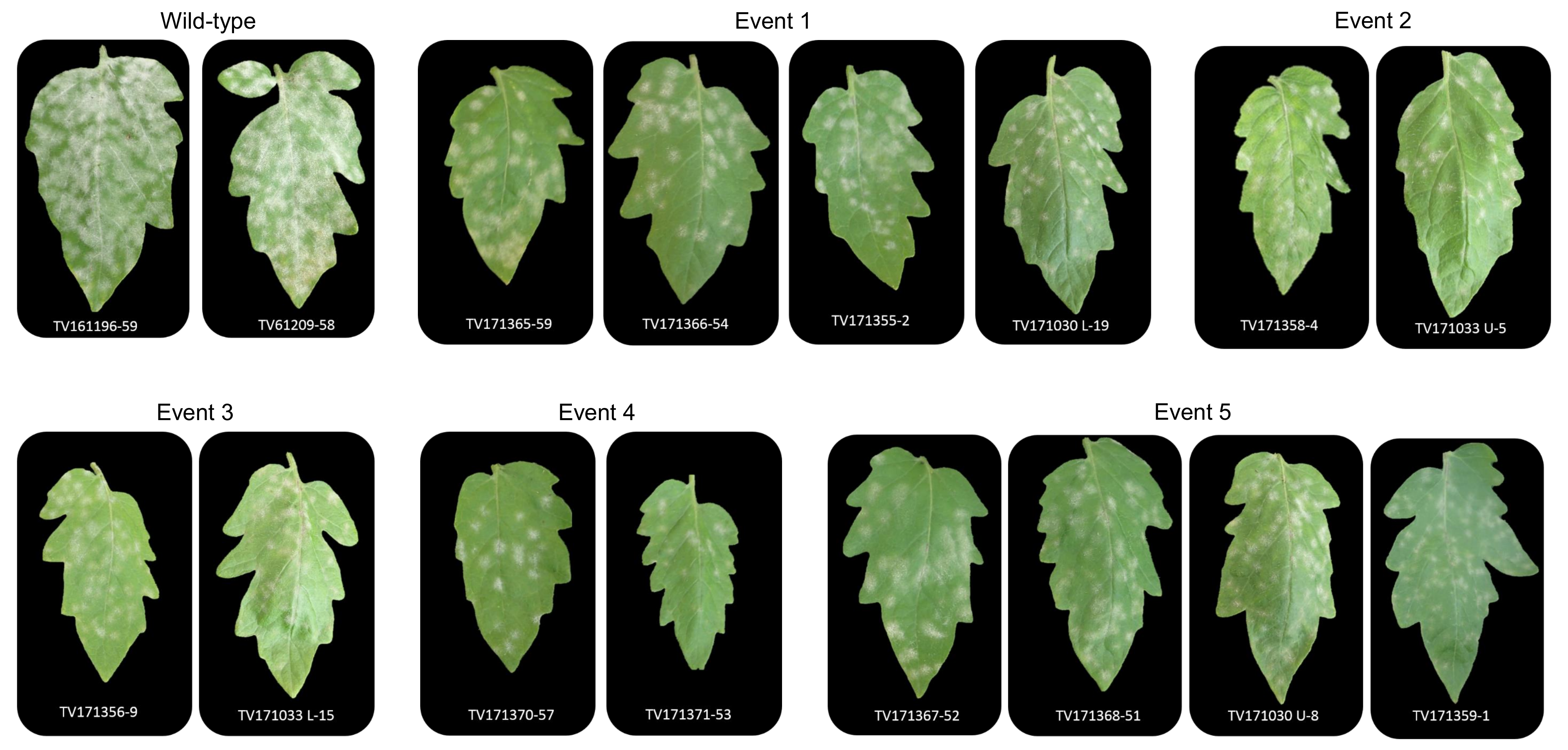

Supplement: Supplementary file 3 — Additional file 3: Supplementary Figure 2. Panel of phenotypes upon infection with Oidium neolycopersici. Leaves from wild-type allele-carrying controls and individual plants of the different slpmr4 mutation classes are shown. Heavy fungal sporulation is present on the leaves of the wild-type plants, while less infection is seen on the leaves of the mutant plants. [file 12870_2020_2497_MOESM3_ESM.tif]

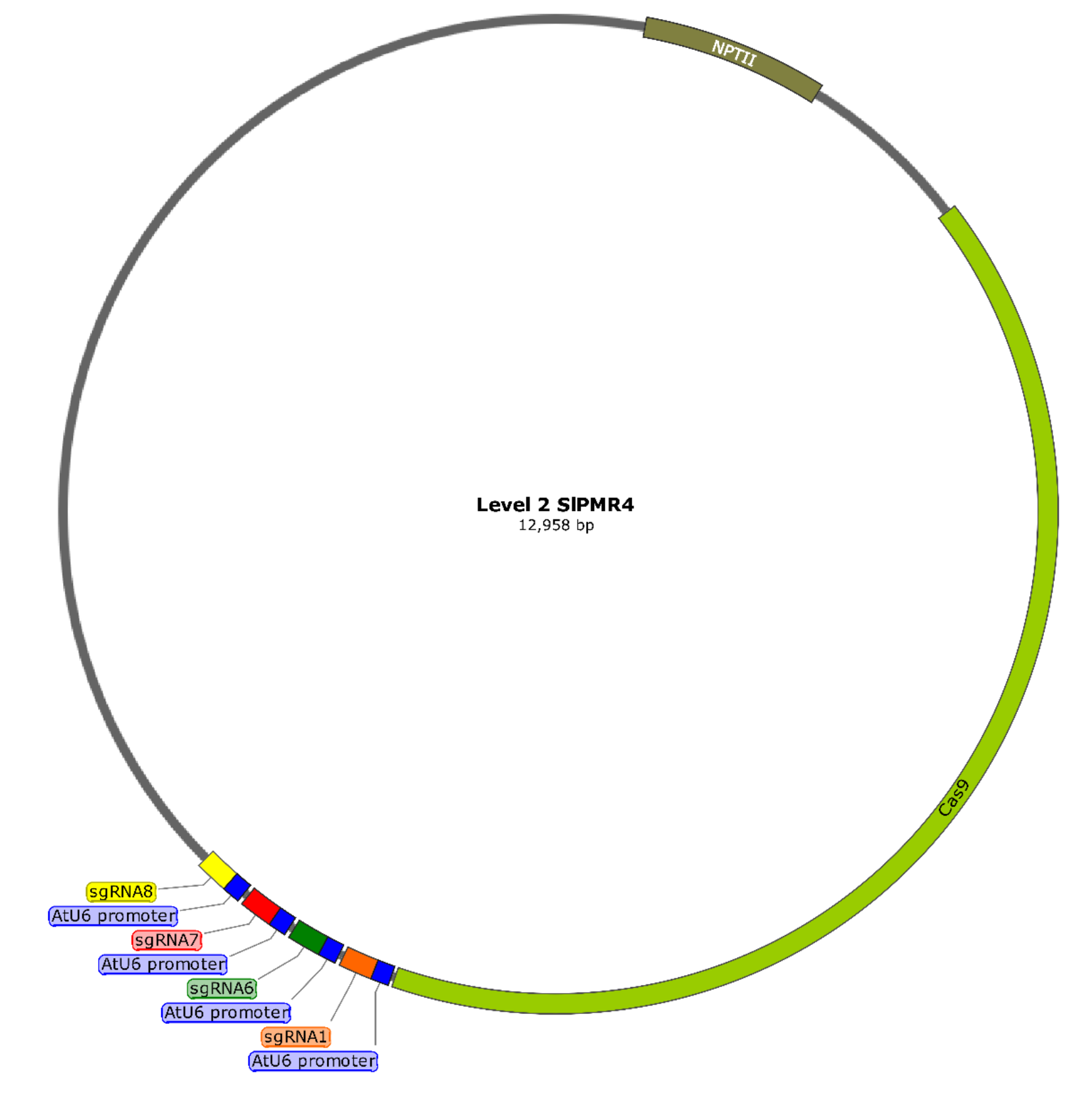

Supplement: Supplementary file 4 — Additional file 4: Supplementary Figure 3. Map of the level 2 vector for CRISPR/Cas9 transformation. The NPTII, Cas9, the four sgRNAs and AtU6 promoters are highlighted. [file 12870_2020_2497_MOESM4_ESM.tif]
